# Supplementary material for: Identification of senescence-associated drivers of tumour growth and progression using a novel microarray platform
Source: bioRxiv. 2025 Dec 23:2025.12.20.695739. Preprint. [Version 1] doi: 10.64898/2025.12.20.695739 (PMC12767529; doi:10.64898/2025.12.20.695739)
Supplement: Supplement 1 [file media-1.pdf]

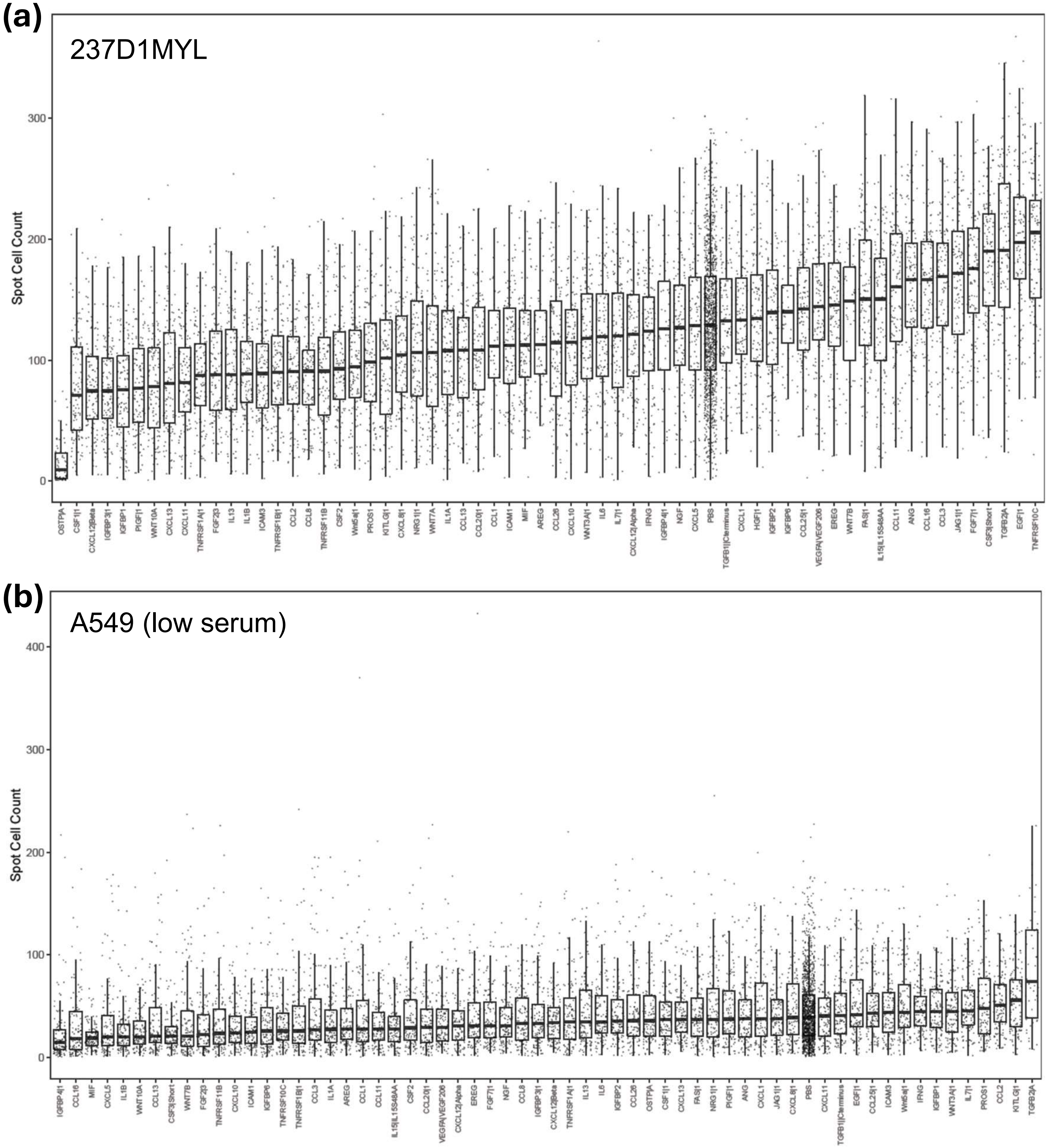

Supplemental Figure 1. Spot cell count for 237D1MYL (**A**) and A549 (low serum; **B**) resulting from exposure to different ligands.

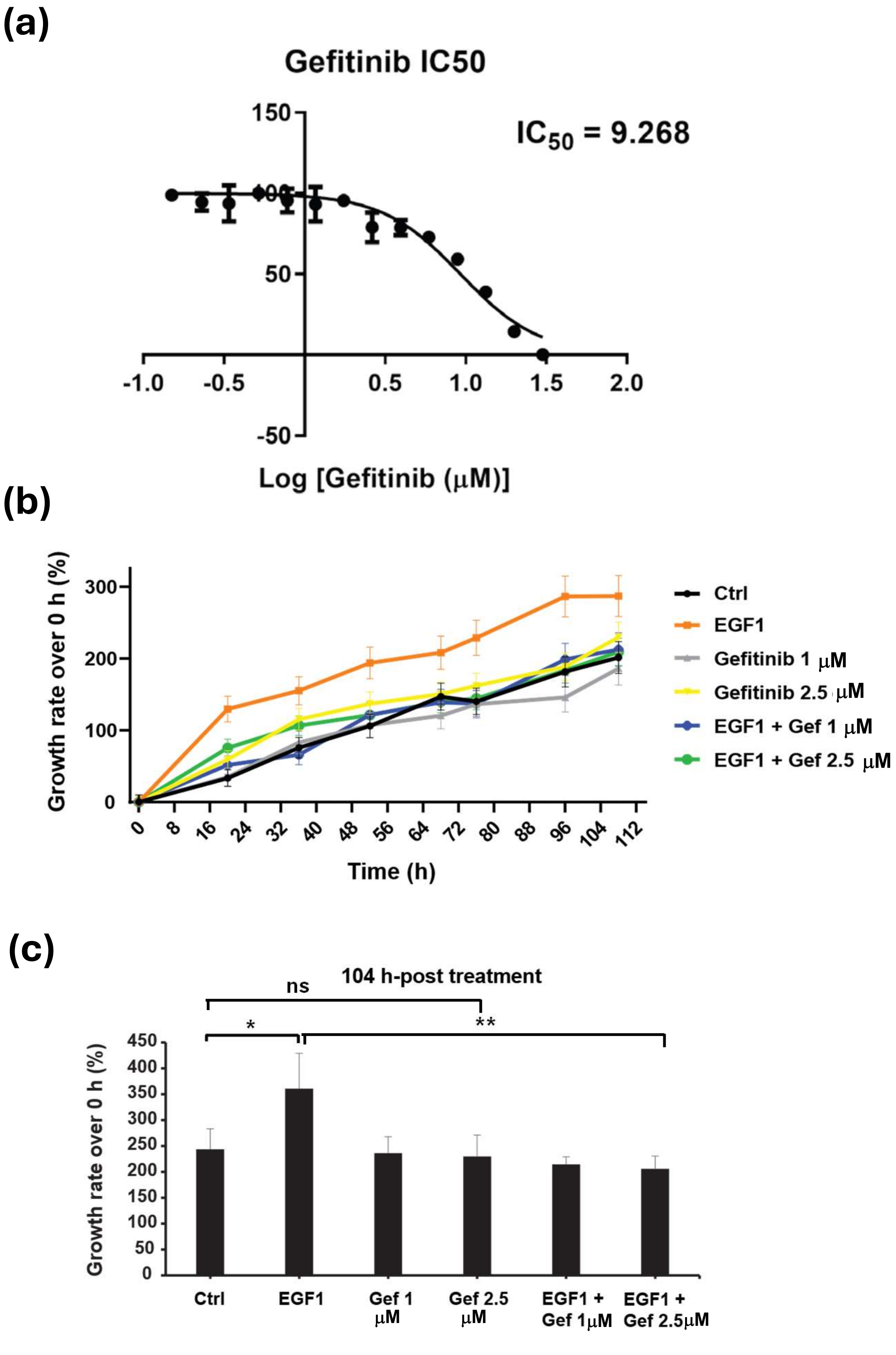

Supplemental Figure 2. Effect of the EGFR inhibitor gefitinib on A549 cell growth. (**A**) A549 cells show limited response to gefitinib over the course of 3 days, with an IC_{50} of 9.268 μM. (**B**) A549 cells treated with EGF show enhanced growth that is reversed by treatment with gefitinib (1 or 2.5 μM); in contrast, these doses of gefitinib have no significant effect on A549 in the absence of EGF. Representative data of 3 independent biological repeats are shown as mean ± SD. (**C**) The growth rate at 104 h-post treatment are shown in bar chart. One-way ANOVA + Tukey HSD test.

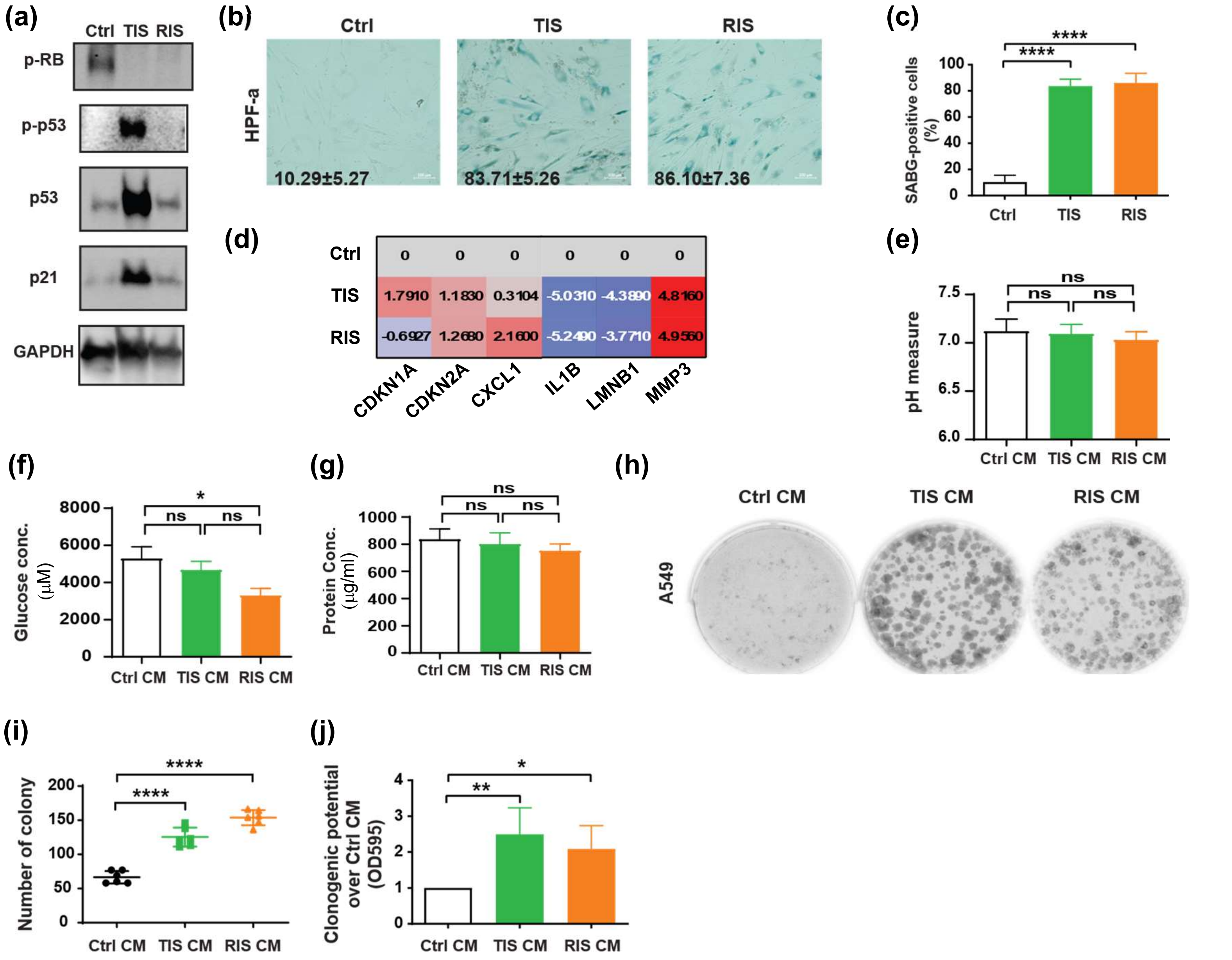

Supplemental Figure 3. Characterization of cellular senescence in HPF-a upon treatment with cisplatin or ionising radiation (IR) and corresponding conditioned media (CM). (a) Protein expression of senescence markers in normal HPF-a or senescent HPF-a induced with cisplatin (TIS, therapy-induced senescence) or IR (RIS, radiation-induced senescence). (b-c) Representative images (b) and quantification (c) of HPF-a stained for SA-b-Gal. A549 cells were untreated (Ctrl) or treated with cisplatin or IR for senescence induction. Cells were fixed on Day 10 after senescence induction and subjected to SA-b-Gal staining using Senescence b-Galactosidase Staining Kit (#9860, Cell Signaling). Images were taken using Zeiss Axio Observer 7 microscope and ZEN Blue software (ver 3.1). Scale bar, 100 mm. Data are shown as mean ± SD from 5 biological replicates. (d) Transcriptional expression of senescence-associated genes detected using quantitative real-time PCR. RNA of A549 cells was extracted on Day 10 after senescence induction and subjected to RT-qPCR. The heatmap are shown as mean fold change in $\log_2$ compared to Ctrl from 3-5 biological replicates. (e-g) Characterization of 72-h CM from Ctrl, TIS, or RIS HPF-a in terms of the pH value (e), glucose concentration (f), and protein levels (g). (h-j) Growth promotion of A549 cells by senescent CM. Colony formation assay was performed to evaluate the impact of senescent CM on the growth of A549. Cells were seeded at a density of 750 cells per well of 6-well plate one day before the treatment. CM treatment was refreshed every 2-3 days throughout the 15-day assay. Representative images (h) and quantification (i-j) of colony formed from 4 biological replicates are shown as mean ± SD. *$p < 0.05$; ** $p < 0.01$; **** $p < 0.0001$; ns, not significant.

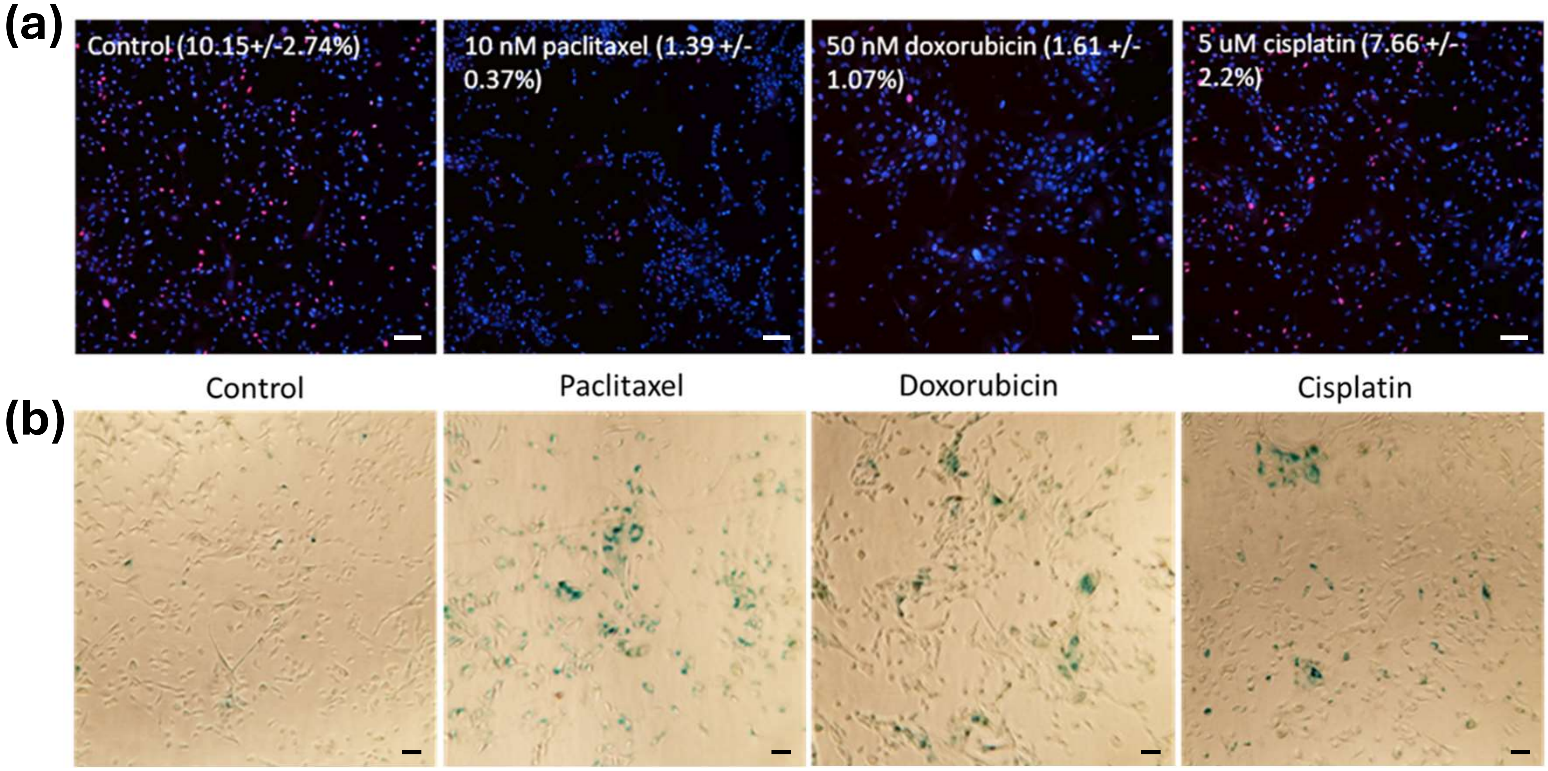

Supplemental Figure 4. Induction of senescence through treatment with chemotherapeutic agents. **A**. Induction of senescence with chemotherapy treatment in the HMEC cell strain 237D1MYL as measured by loss of EdU incorporation or **B.** SABgal staining (**B**). Values in **A** represent percentage of cells positive for EdU incorporation, +/- S.D. from triplicate wells. Scale bar in A is 100 µm and scale bar in B is 50 µm.

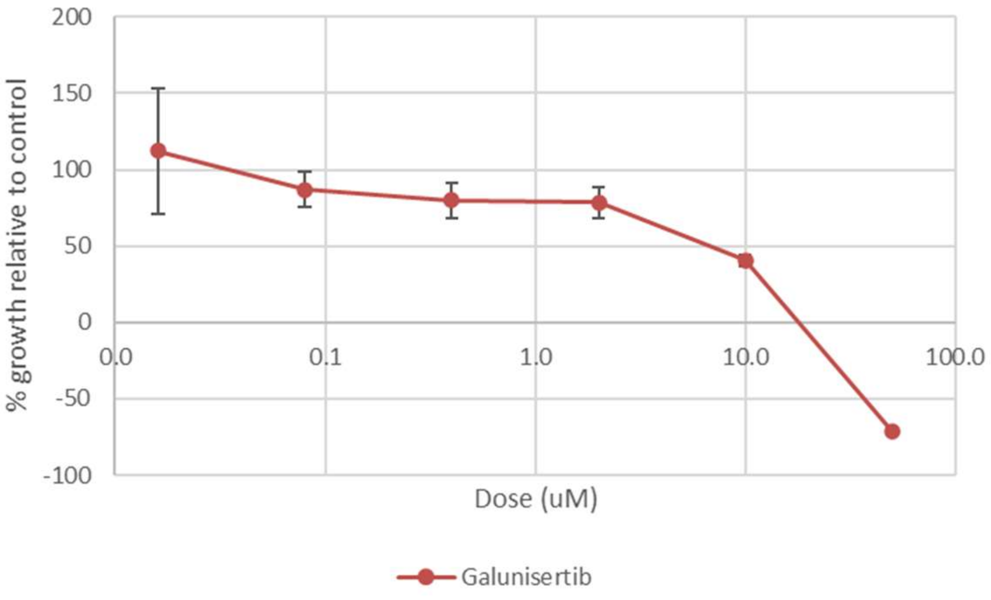

Supplemental Figure 5. Effect of the TGFBR inhibitor galunisertib on HMEC 237D1MYL cell growth.

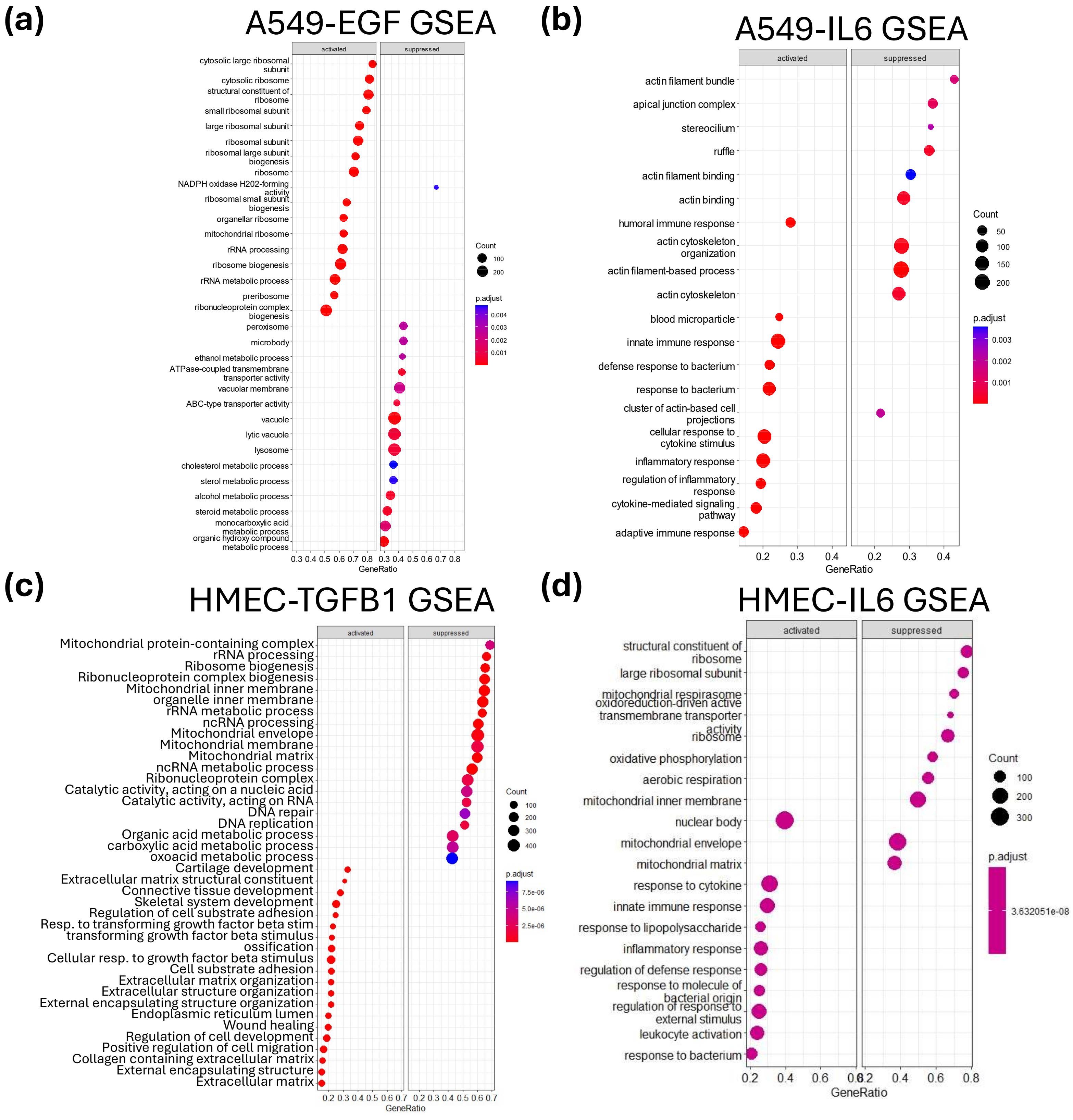

Supplemental Figure 6. GO GSEA analysis of responses to treatment in lung cancer and breast HMEC cells. (**A**) Dotplot of gene sets that were activated or repressed post treatment of A549 cells with EGF1. (**B**) Dotplot of gene sets that were activated or repressed post treatment of A549 cells with IL6. (**C**) Dotplot of gene sets that were activated or repressed post treatment with TGFB1 in the HMEC. (**D**) Dotplot of HMEC gene sets that were activated or repressed post treatment with IL-6 in the HMEC.
